# Diagnostic significance of atypical category in the voided urine samples: A retrospective study in a tertiary care center

**DOI:** 10.4103/0974-7796.68857

**Published:** 2010

**Authors:** Ghadeer A. Mokhtar, Mohamed Al-Dousari, Doaa Al-Ghamedi

**Affiliations:** Pathology Laboratory, King Abdul-Aziz University Hospital, Jeddah, Saudi Arabia

**Keywords:** Atypical urine, atypical urothelial cells, urine cytology, urothelial carcinoma, voided urine

## Abstract

**Background:**

Voided urine samples continue to play an important role in the surveillance of urothelial malignancy and also as a screening mode for high risk patients. In some cases, it is difficult to reliably distinguish changes induced by inflammation, stone or other reactive condition from neoplasm, and these cases are categorized as atypical. The aim of our study is to evaluate the prevalence and the significance of atypical diagnosis in the voided urine samples and also to identify the cytomorphologic features that are seen more frequently in the atypical malignant urine samples.

**Materials and Methods:**

All voided urine cytology samples with a diagnosis of atypical urothelial cells, between the period of 2000 and 2009, were obtained from the cytology database. Only those cases with histologic follow-up were included in the study. The cytology and the histology slides were retrieved and reviewed. The following parameters were evaluated: cellularity, cell clusters, nuclear membrane irregularities, hyperchromasia and India-ink type nuclei, the presence of spindle cells and the cytoplasmic characteristics.

**Results:**

Out of 72 voided urine samples included in the study, 49 cases (68%) had a positive histologic diagnosis of urothelial malignancy in the follow-up histology; of these (55%) were high-grade urothelial carcinoma. Increased cellularity, papillary cell clusters, nuclear membrane irregularity, hyperchromasia and India-ink type nuclei were observed more frequently in the atypical malignant urine samples, while cytoplasmic vacuolization were seen more in the negative reactive urine samples.

**Conclusion:**

The atypical category diagnosis is associated with a significant proportion of urothelial carcinoma. It should be used by the pathologist to convey concern to the clinician in difficult cases that may require close follow-up.

## INTRODUCTION

Urine examination is considered to be one of the oldest clinical laboratory tests known to humans. The examination of urine sediment smears was first popularized by George Pananicolaou and Marshall in the 1940s for bladder cancer detection and follow up.[1] Indications for urine cytology fall mainly into three categories; the most common one is patients with hematuria. The second indication is follow-up of patient with bladder cancer and third is as screening of high-risk groups for bladder cancer such as those exposed to aniline dye or to aromatic amines and those with history of urinary bilharziasis. The accuracy of urine cytology diagnosis depends on several factors that are related to tumor grade, type of the specimen and sampling. It has been widely accepted for the diagnosis of high-grade urothelial carcinoma with a sensitivity as high as 98%.[2] However, low-grade tumors are not detected reliably by cytology, with sensitivity and specificity values as low as 8.5 and 50%, respectively.[3] In addition, specimen type can also affect the interpretation of urine cytology, with voided specimens being more specific but slightly less sensitive than instrumented urine.[3] This in fact could be explained by the absence of the instrumentation-induced reactive changes. Finally, it has been shown by several studies that increasing the number of the samples will increase the sensitivity of urine cytology, especially for the detections of high-grade lesions.[4,5] There are several situations that can affect the cellularity and the cytology of the cells, including instrumentation, inflammation, infection, surgical manipulation, treatment with chemo and radiotherapy and calculi, making it difficult even for the experts to reliably discriminate malignant cells.[6,7] These cases often fall into the atypical categories. However, there is lack of consensus regarding the terminology and the diagnostic criteria that should be used for urothelial atypia and the “atypical” category remains a wastebasket diagnosis that is used variably by individual cytopathologists in different institutions. In 2004, the Papanicolaoau Society of Cytopatholgy recommended to include “atypical urothelial cells” as a diagnostic category in the urine cytology, with a comment in the report to further classify the atypia as reactive or neoplastic.[8] However, the criteria to separate reactive from neoplastic atypia are not well defined in this article or in the literature, in general. Thus, in the absence of agreement and the lack of diagnostic criteria for urothelial atypia, the atypical urothelial cell category remains one of the challenging diagnostic entities.

Therefore, the aim of this retrospective study is to evaluate voided urine samples reported as atypical and to assess the clinical significance of this category through histologic correlation of these samples. In addition, we assessed the cytologic features of the atypical urine samples and compared the ones with positive follow-up to those with negative follow-up.

## MATERIALS AND METHODS

From the cytology information system, we retrieved all the cytologic voided urine specimens received from the year 2000 to the end of the year 2009, with the diagnosis of atypical urothelial cells. We then searched for all of the subsequent surgical follow-ups of these specimens and retrieved them with their reports and clinical information such as age, gender and previous samples. There were 260 voided urine samples that have been categorized as atypical; of these only 76 cases had histologic follow up. The cytologic diagnoses were categorized into atypical, not otherwise specified, atypical favoring reactive and atypical favoring neoplasm. This subclassification is based on parameters which include cellularity, cell clusters and nuclear features. Cellularity was graded as low cellularity with an average of 12 cells per low-power field, moderate cellularity with an average of 12–30 cells per low-power field and high cellularity with more than 20 cells per low-power field. A cluster was defined by the presence of three or more cohesive transitional cells. Nuclear features included enlarged nucleus with high nuclear-to-cytoplasmic ratio, hyperchromasia, irregularity of the nuclear membrane and prominent nucleoli.

On reviewing the cytologic specimens, urothelial cells exhibiting a nuclear/cytoplamic (N/C) ratio of more than 50% is considered atypical in voided urine.[9] The atypical favoring reactive is reserved for the atypical cases, in which the cells are present in clusters and have bubbly cytoplasm but with intact, smooth nuclear membrane and small conspicuous nucleolus.

The atypical, not otherwise specified, is used when urothelial cells, even if single, appeared degenerated but displayed a high N/C ratio, intact but irregular nuclear membranes with clump chromatin. These cells that are degenerated but have irregular nuclear membrane have been reported by numerous authors to be associated with high-grade urothelial carcinoma.[10,11]

The atypical favoring neoplasm diagnosis is reserved for cells with nuclear features, cell clustering and mild to moderate increase in cellularity.

For the cytologic-histologic follow-up and correlation, histology was considered the “gold standard”. An arbitrary period of 1 year was selected as the maximal interval allowed between cytology and histology to assess concordance.

On reviewing the histologic specimens, the World Health Organization (WHO/ISUP) classifications were used for categorizing the revised slides.[12] It recognizes a rare benign papilloma, a group of papillary urothelial neoplasm, as low malignant potential and two grades of carcinoma (low and high grade).

The histopathologic diagnosis is then categorized into two main groups: the positive category that includes papillary urothelial neoplasm of low-malignant potential (PUNLMP), low-grade urothelial carcinoma (LGUCA) and high-grade urothelial carcinoma (HGUCA). The second group is the negative one which includes cystitis with atypia and urothelial atypia of undetermined significant. When more then one cytologic specimen existed in the cytohistologic follow up and correlation, the worst was used as long as it was rendered within 1 year of the follow-up biopsy.

The percentage of each category and their subsequent follow up was calculated to determine the significance of the atypical category in the voided urine sample.

In addition, the detailed cytologic features such as cellularity, cell clustering, papillae, nuclear membrane abnormalities, chromatin pattern, nuclear pleomorphism, India-ink nuclei, spindle cells and cytoplasmic detail were compared between the negative and the positive groups.

## RESULTS

### Clinical data

The age range of patients was between 29 and 82 years with a mean of 42 years and a median of 41. The majority of patients were males (64 patients, 89%). There were 55 (76%) patients who presented with hematuria for investigations while the remaining the voided urine samples were submitted as a screening. None of the patients had a cystoscopy prior to the urine samples.

### Cytology review

Out of 1250 voided urine samples seen during the period between 2000 and 2009, the atypical category diagnosis constituted 260 cases (21%). Only 76 cases (29%) had subsequent biopsy and, therefore, histologic follow up. These 76 cases were selected for review.

The 76 cases were diagnosed previously as follows: 49 cases (65%) as atypical urothelial cells, not otherwise specified (NOS) [Figure 1a], 13 cases (17%) as atypical urothelial cells favoring reactive [Figure 2a] and 14 cases (18%) as atypical urothelial cells favoring neoplasm [Figures 3a and 4a]. Upon review; two cases diagnosed as atypical cells favoring reactive were reclassified as negative, one case diagnosed as atypical urothelial cells favoring neoplasm was revised into high-grade urothelial carcinoma and one case was revised into atypical squamous cells. These four cases were excluded from the study. Of the 49 cases diagnosed as atypical urothelial cells, NOS, 27 cases were revised into atypical urothelial cells favoring neoplasm. [Table 1] summarizes the diagnostic subcategories of atypical urine and the revised diagnosis upon review.

**Figure 1a:**
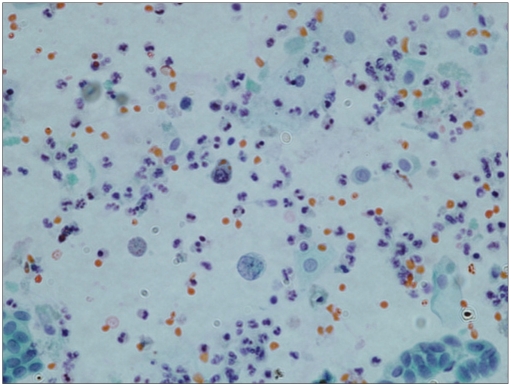
Scattered single atypical but degenerated urothelial cells with India-ink type nuclei in a background of extensive inflammation (Papanicolaoau stain, ×600)

**Figure 2a:**
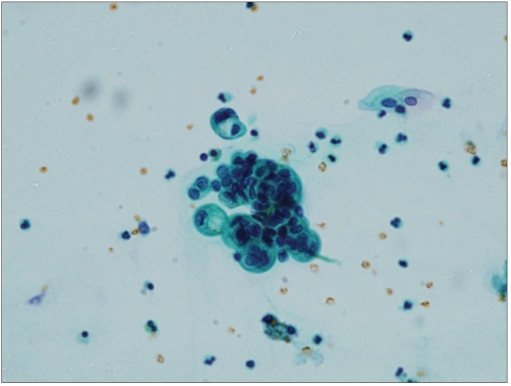
A case of atypical urothelial cells favoring reactive showing crowded, hyperchromatic groups of urothelial cells with focal cytoplasmic vacuolization (Papanicolaoau stain, ×600)

**Figure 3a:**
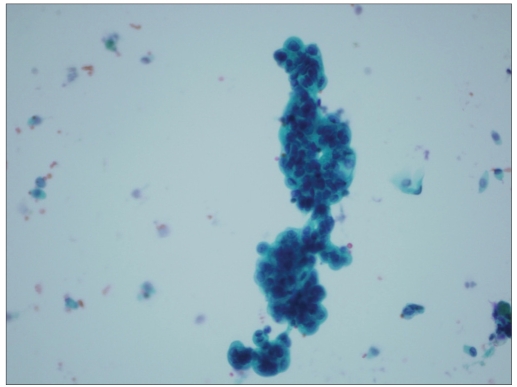
Pseudo-papillary clusters of crowded, hyperchromatic groups of urothelial cells; this case was diagnosed as atypical urothelial cell favoring neoplasm (Papanicolaoau stain, ×400)

**Figure 4a:**
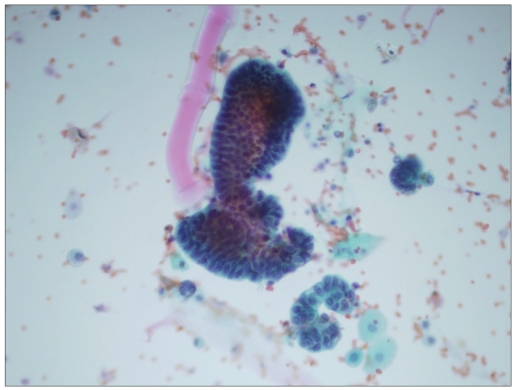
Papillary clusters of crowded, hyperchromatic urothelial cells favoring neoplasm (Papanicolaoau stain, ×400)

**Table 1:** Diagnostic categories of 76 cases of atypical voided urine

Initial cytology diagnosis	Revised cytology diagnosis
Atypical urothelial cells, NOS (n=49)(65%)	Same (n=21) (44%) Atypical urothelial cells, favor neoplasm (n=27) (54%) Atypical squamous cells (n=1) (2%)
Atypical urothelial cells, favor neoplasm (n= 14) (18%)	Same (n=13) (93%) HGUC (n=1) (7%)
Atypical urothelial cells, favor reactive (n=13) (17%)	Negative (n=2) (15%) Same (n=11) (85%)

NOS - Not otherwise specified; HGUC - High grade urothelial carcinoma

A majority of the specimens (46%) showed (3+) cellularity, and of these 64% were in the category of atypical favoring neoplasm. Cell clusters were noted in 60 cases (83%); 39 (65%) of these were in the category of atypical favoring neoplasm. Of these clusters, papillae were seen in 45 cases (62.5%); 85% were in the category of atypical favoring neoplasm. India-ink type nuclei were present in only six cases; four of them were classified as atypical favoring neoplasm (83%). Nuclear membrane irregularities and hyperchromasia were observed more frequently in the category of atypical favoring neoplasm, constituting 55 and 84%, respectively. Cytoplasmic vacuoles were present more frequently in the category of atypical favoring reactive (41%). Spindle cells were noted in only three cases (4%). Table 2 summarizes the distribution of the cytologic features among the different categories of atypical urine samples.

**Table 2:** Distribution of the cytological features within the subgroups of atypical urine cytology

Cytological features	No. of patients	No. of atypical favor reactive	No. of atypical, NOS	No. of atypical favor neoplasm
Cellularity				
1+	14 (19)	6 (43)	3 (21)	5 (36)
2+	25 (35)	4 (16)	7 (28)	14 (56)
3+	33 (46)	1 (3)	11 (33)	21 (64)
Cell clusters	60 (83)	6 (10)	15 (25)	39 (65)
Papillae	45 (62.5)	2 (4)	5 (11)	38(85)
India ink-type nuclei				
Present	6 (8)	0	2 (33)	4 (67)
Absent	66 (92)	11(17)	19 (29)	36 (54)
Nuclear membrane irregularities				
Present	58 (81)	9 (16)	17 (29)	32 (55)
Absent	14 (19)	2 (14)	4 (29)	8 (57)
Hyperchromasia				
Present	38 (53)	1 (3)	5 (13)	32 (84)
Absent	34 (47)	10 (29)	16 (47)	8 (24)
Cytoplasmic vacuoles	27 (37.5)	11 (41)	6 (22)	10 (37)
Spindle cells	3 (4)	0	1 (33)	2 (67)

NOS - Not otherwise specified; Figures in parenthesis are in percentage

### Cytology-histology correlation

Upon reviewing the slides of the biopsies and correlating the diagnosis with the corresponding cytologic diagnosis for the 72 cases included in the study, we obtained the following results.

All the 11 urine samples with the diagnosis of atypical urothelial cell, favored reactive (100%) and 12 out of 22 cases (57%) of the atypical urothelial cells, NOS, had negative histology on the subsequent biopsies. The histologic diagnosis of these negative cases was as follows: three cases of hyperplastic urothelium, two cases of atypical urothelium of undetermined significance [Figure 3b], eight cases of cystitis with reactive atypia [Figure 2b] and 12 cases had no significant pathological diagnosis.

**Figure 3b:**
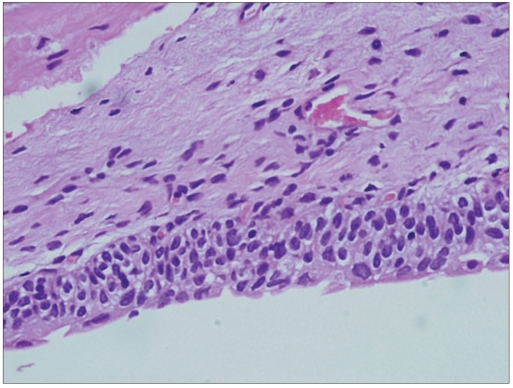
Histology of this case showed a hyperplastic urothelium with mild atypia of urothelial cells (H and E, ×600)

**Figure 2b:**
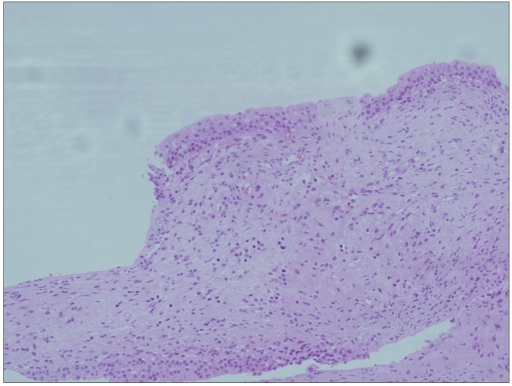
Follow up of this case showed chronic cystitis with reactive urothelial cells (H and E, ×200)

The remaining 49 (68%) specimens had a positive histologic diagnosis for malignancy. Within this group of patients, there were 27 (55%) patients with high-grade urothelial carcinoma [Figure 1b] and 19 49 (39%) patients with low-grade urothelial carcinoma [Figure 4b]. One patient (2%) had a papillary urothelial neoplasm of low malignant potential.

**Figure 1b:**
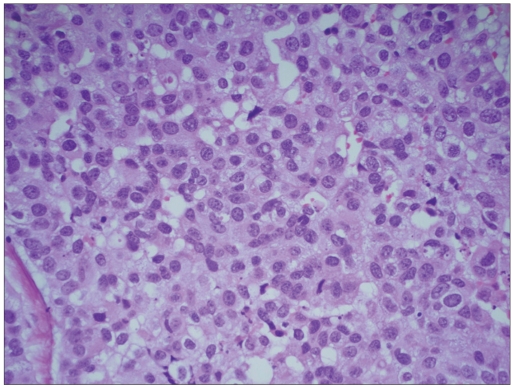
Histology showed high-grade urothelial carcinoma (H and E, ×400)

**Figure 4b:**
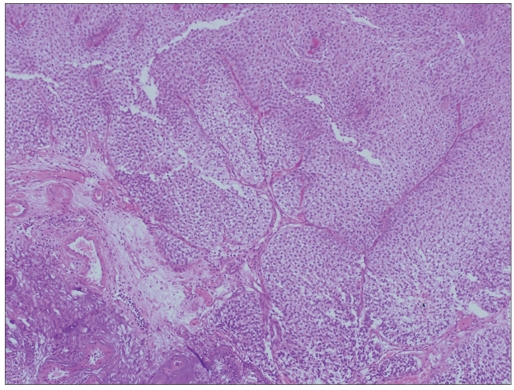
Follow-up histology is low-grade urothelial carcinoma (H and E, ×200)

Table 3 summarizes the cytologic-histologic correlation of the 72 cases according to the diagnostic categories.

**Table 3:** Cytologic-histologic correlation of 72 atypical voided urine included in the study

Cytology diagnosis	Negative histology N (%)	PUNLMP/LGUCA N (%)	HGUCA N (%)
Atypical urothelial, favor reactive (n=11)	11 (100)	-	-
Atypical urothelial cells, NOS (n=21)	12 (57)	2 (10)	7 (33)
Atypical urothelial cells, favor neoplasm (n=40)	2 (5)	18 (45)	20 (50)
Total (n=72) (100%)	25 (34.5)	20 (28)	27 (37.5)

NOS - not otherwise specified; PUNLMP - Papillary urothelial neoplasm of low malignant potential; LGUCA - Low grade urothelial carcinoma; HGUC - High grade urothelial carcinoma

More cellularity was noted in the specimen that had a positive cytologic follow up, with 57% of these being 3+. Cell clusters were seen in 60 cases (83%) and were more frequent in the specimen that had urothelial malignancy in the follow up (70%); of these, 45 cases had papillary configuration [Figures 3a and 4a]. India-ink nuclei were present in only six cases (8%); five (83%) had a positive histologic follow up [Figure 1a]. Nuclear membrane irregularities and hyperchromasia were also seen more frequently in the cases that had urothelial cell carcinoma in the biopsy materials, i.e., 69 and 89%, respectively. Cytoplasmic vacuoles were seen mainly in cases with reactive urothelial changes (67%), and also in 33% of positive cases. Spindle cells are seen only in cases that were positive for urothelial carcinoma in the follow up [Table 4].

**Table 4:** Correlation of the cytological features with the outcome

Cytological features	No. of patients	Negative for UC (n=25)	Positive for UC (n=47)
Cellularity			
1+	14 (19)	9 (64)	5 (36)
2+	25 (35)	10 (40)	15 (60)
3+	33 (46)	6 (18)	27 (82)
Cell clusters	60 (83)	18 (30)	42 (70)
Papillae	45 (62.5)	10 (22)	35 (78)
India ink-type nuclei			
Present	6 (8)	1 (17)	5 (83)
Absent	66 (92)	24 (36)	42 (64)
Nuclear membrane irregularities			
Present	58 (81)	18 (31)	40 (69)
Absent	14 (19)	7 (50)	7 (50)
Hyperchromasia			
Present	38 (53)	4 (11)	34 (89)
Absent	34 (47)	21 (62)	13 (38)
Cytoplasmic vacuoles	27 (37.5)	18 (67)	9 (33)
Spindle cells	3 (4)	0	3 (100)

UC - Urothelial carcinoma; Figures in parenthesis are in percentage

## DISCUSSION

Urine cytology is used for the primary and recurrent diagnosis of urothelial carcinoma. The sensitivity and specificity vary according to the collection methods and tumor grade.[3] However, the overall specificity for transitional cell carcinoma (TCC) is more than 90%. False positive results occur in association with stone, human polymavirus infection and chemotherapy effect.[3] False negative results occur due to inability to sample some lesions or difficulties in diagnosing some entities like low-grade cancer.[3]

The atypical category in the urine cytology still remains a wastebasket and includes both the specimens that have a significant lesion and others that do not have.[13] In addition, most of the physicians are confused with regard to the treatment/follow-up strategies, when faced with this diagnosis, and a significant number of patients who have specimens labeled as atypical are not biopsied.

Renshaw attempted to categorize atypical urine specimens based on the cytomorphologic features and the risk associated with each group. Diffuse cellular atypia and India-ink type nuclei were identified as features associated with neoplasia, and it was suggested that the presence of cell clusters, reactive changes, diffuse mild atypia and focal degenerative changes should be ignored.[13]

In a recent study by Raab *et al*.,[3] they reported an atypical voided urine specimen at a rate of 10.1%. This is in contrast to a rate of only 1.9% in a study by Bhatia *et al*.[14] The rate of atypical urine diagnosis in voided specimens at our institution is 16%.

In our study, we evaluated voided urine samples because they continue to be used as a screening tool for TCC. At our hospital, a good number of patients who have urine specimens that are labeled as atypical are not biopsied. In an attempt to provide an accurate result, we perform our study by selecting a patient population with follow-up biopsy. We found a high percentage (65.5%) of atypical urine that subsequently showed TCC on biopsy. Of these positive cases, the majority had high-grade urothelial carcinoma (37.5%). Malignancy rate as a follow up of atypia diagnosis in the voided urine ranged from 23.3 to 68% in different studies.[3,7,13,14] This wide range reflects the difficulty in defining this category precisely leading to great interobserver variability among cytopathologists. In the current study, the follow-up histology in the majority of the cases was HGUC (37.5%). This is different from the result obtained by Deshpande and Mckee,[7] which showed the majority of their cases to be LGUC, but similar to what was observed by Kapur *et al*.[6] This may be because of the different classification systems adopted by the different investigators.

It is interesting in this study that none of the patients with atypical favor reactive diagnosis in the urine samples had urothelial carcinoma on the follow-up biopsy. However, in a study by Brimo *et al*., of 282 urine samples the rate of malignancy among this category was 29%.[9]

The significance of cell clusters in voided urine is a controversial issue. In one study, tissue fragments were significantly more common in urine specimens from patients who had urothelial carcinoma on the follow-up histology than from patients who had negative biopsy results.[15] Other studies had failed to reproduce similar results.[16] However, in a more recent study by Deshpande *et al*., of the 201 voided urine samples, the number of the cell clusters was associated significantly with the presence of TCC.[7] In the current study, cell clusters were noted in voided urine specimens from both negative and positive biopsies but were more frequent in those who had urothelial carcinoma in the follow up (70%). Of these, papillae were more frequently noted in voided urine samples from patients with positive histology for TCC (74%) than those with negative histology (40%).

Alterations in nuclear morphology were most consistently observed in malignant atypical cells; these include hyperchromasia, nuclear pleomorphism and nuclear membrane irregularities.[10,17] In the current study, these cytologic findings were observed more frequently in malignant atypical smears and rarely observed in few benign cases.

India-ink type nuclei (also termed coal black nuclei) had been shown by Renshaw and other authors in their studies to be significantly associated with TCC on the follow up.[13] In our study, India-ink type nuclei were seen in only 8% of specimens; however, 83% of these were associated with positive histology for TCC in the follow up.

Spindle cells were observed in only three cases in our study (4%); however, all these three cases showed high-grade transitional cell carcinoma in the biopsy material. This is similar to what have been reported in other previous studies.[7,18]

In conclusion, the atypical diagnostic categories in reporting urine cytology samples should not be categorized with the negative group. This important category should be used in difficult cases that may require close follow up and there should be clear communication between the cytopathologist and the clinician with regard to the meaning of atypia, in order to avoid missing significant lesions. In addition, using definitive criteria for evaluating urine specimen would increase the sensitivity and the accuracy of the cytology diagnosis.
